# Comparative Analysis of Sponge-Associated, Seawater, and Sediment Microbial Communities from Site F Cold Seep in the South China Sea

**DOI:** 10.3390/microorganisms13122672

**Published:** 2025-11-24

**Authors:** Yan Wang, Lin Gong, Zhaoming Gao, Dong Dong, Xinzheng Li

**Affiliations:** 1 Department of Marine Organism Taxonomy & Phylogeny, Qingdao Key Laboratory of Marine Biodiversity and Conservation, Institute of Oceanology, Chinese Academy of Sciences, Qingdao 266071, China; 2 University of Chinese Academy of Sciences, Beijing 100049, China; 3 Institute of Deep-Sea Science and Engineering, Chinese Academy of Sciences, Sanya 572000, China; 4 Laboratory for Marine Biology and Biotechnology, Qingdao National Laboratory for Marine Science and Technology, Qingdao 266000, China

**Keywords:** site F cold seep, microbiome, metagenome, chemolithoautotrophic symbiosis

## Abstract

Microbial communities at Site F cold seep, ubiquitous in both the environment and the associated fauna, demonstrate clear habitat-specific partitioning. Metagenomic sequencing and binning demonstrated a striking partitioning of microbial taxa at the cold seep: whereas the sponge-associated microbiome was distinctly enriched with specialized sulfur- and methane-oxidizing bacteria that were rare in the environment, it simultaneously exhibited a significantly reduced archaeal content, lower α-diversity, and a simpler overall community structure compared to the sediment and seawater communities. Distinct evolutionary lineages and varying abundances were observed among the microbiomes from seawater, sediment, and sponges. Furthermore, their Metagenome-Assembled Genomes (MAGs) exhibited significant differences in genomic features, including genome size and GC content. The sponge-associated microbiome exhibits lower diversity but maintains a high abundance of key functional genes, particularly those involved in sulfur cycling (e.g., *apr*, *dsr*, *metZ*), indicating enhanced metabolic efficiency in energy conservation and nutrient acquisition. This study reveals that the seawater, sediment, and sponge-associated microbiomes exhibit genome simplification and functional specialization in the cold seep environment, with varying lifestyles driving structural optimization and functional remodeling of the symbiotic microbiomes.

## 1. Introduction

Cold seep ecosystems, as representative examples of deep-sea ecosystems, sustain a significant portion of their ecosystem through the utilization of chemical energy and have been extensively studied worldwide. Cold seeps typically refer to the emergence of chemical-rich fluids from below the seafloor, along specific pathways under particular temperature and pressure conditions. Therefore, through the release of fluids such as methane—a potent greenhouse gas—cold seeps play a critical role in carbon cycling and are a major focus of global change research. The physical and chemical environment of cold seeps provides essential substrates for chemolithoautotrophic organisms, whose proliferation transforms these areas into key hotspots for deep-sea biological interactions [1].

China has preliminarily identified a total of seven offshore cold seep areas, with six of them distributed in the South China Sea. Over 40 cold seep carbonate sediment stations have been reported in this region [2]. Among these, the Site F cold seep [3,4] and the Haima cold seep [5] are noted as the most active modern cold seep regions in the South China Sea. The active Site F cold seep is located at the summit of the southern segment of the Formosa Ridge, measuring approximately 90 m in length and 80 m in width, primarily composed of authigenic carbonates [2]. Based on the monitoring of the environmental characteristics at the site F cold seep, the depth is approximately 1120 m, with water temperatures around 3.4 °C at the seawater-sediment interface, a pH of about 7.7, and a salinity of approximately 34.5. The maximum current velocity reaches up to 0.35 m/s. The concentrations of carbon dioxide and dissolved oxygen are relatively stable, averaging 815 ppm and 3.07 mg/L, respectively, while methane concentrations exhibit significant variability, fluctuating between 237.25 ppm and 15,000 ppm [6].

At the F Site cold seep, large benthic fauna has been identified, comprising at least 41 species or taxa from seven phyla: Porifera, Cnidaria, Mollusca, Arthropoda, Annelida, Echinodermata, and Chordata [2]. The dominant biomass includes the mussel *Gigantidas platifrons* and the squat lobster *Shinkaia crosnieri*. Major sponge species (Phylum Porifera; Class Hexactinellida) reported at the site comprise *Semperella jiaolongae* and *Euplectellidae* gen. et sp. [7], both belonging to the class Hexactinellida. Due to their unique aquiferous canal systems, sponges engage in extensive and frequent material exchange and reactions with the seawater and sediments through physiological responses such as filtration and sediment adsorption.

The discovery of cold seeps and hydrothermal systems, along with their unique chemiosynthetic environments, has provided ideal conditions for the diversification and specialization of biological communities, significantly expanding our understanding of life activities under extreme deep-sea environmental conditions [8]. Microbial communities in cold seeps, encompassing both free-living and symbiotic forms, rely on chemolithoautotrophic processes to provide essential energy to the cold seep ecosystem. As primary producers within this ecosystem, these microorganisms support the survival of larger benthic organisms such as polychaetes, bivalves, and amphipods [9,10,11,12]. In the F Site cold seep ecosystem, the symbiotic relationships between larger organisms and chemolithoautotrophic microbes are a key survival strategy. The benthic environment here is notably characterized by high-biomass beds of Bathymodiolus platifrons [10]. Their high abundance and density are supported by a symbiosis with methane-oxidizing bacteria, which satisfy most of their nutritional needs and are crucial for this ecological pattern [3,13,14,15].

Research on sponge-microbe symbionts in the extreme environments of deep-sea hydrothermal vents and cold seeps has been challenging due to the randomness and rarity of sampling. Limited studies have confirmed the presence of sulfur-oxidizing bacteria and bacteriophages within sponges, as well as highly specific methanotrophic bacteria, suggesting that this beneficial nutritional symbiosis aids host sponges in adapting to hydrothermal vent environments [16]. Recent research on sponges from *Cladorhiza* and *Spinularia* genus living at hydrothermal vents has also confirmed the presence of potential chemosynthetic deformibacteria, showing carbon and nitrogen isotope characteristics associated with chemolithoautotrophic contributions [17]. Sulfur-oxidizing bacteria appear to be a dominant symbiotic group in sponges within chemosynthetic environments, as previous studies have reported high abundances of the sulfur-oxidizing SUP05 strain in sponges from both cold seep and hydrothermal vent environments, verifying that the genome of SUP05 associated with sponges contains multiple genes related to sulfur oxidation, including *sat*, *aprAB*, *dsrAB*, *soxA*, *soxB*, *soxX*, *soxY*, and *soxZ* [18,19,20]. It is essential to conduct more extensive research on sponge-associated microbiomes in chemosynthetic environments.

Chemosynthetic environments shape distinct patterns in microbial community structure and their potential functions. These geographically consistent differences have been validated across diverse regions globally, including the Cascadia Margin, Hydrate Ridge, Gulf of Mexico, Pacific Ocean Margin, Nankai Trough, Andaman Sea, Ulleung Basin, and the South China Sea [19]. However, with regard to the characteristics of global sponge-associated microbial communities, host specificity is a major feature of these communities worldwide. Although sponges are continuously engaged in activities such as pumping and filtering water, leading to a constant influx of seawater, they still maintain highly diverse and specific microbial communities [20,21,22]. At site F cold seep, we serendipitously discovered a unique assemblage of sponge communities, where different species and individuals coexist in the same area, forming a sponge ground. The sponge individuals adhere to the same substrate and filter the same seawater, making it more likely that the symbiotic microbes within them represent the true sponge-associated microbiome [23]. However, whether the sponge-associated microbiomes from the cold seep sponge ground exhibit differences and potential interactions compared to the ambient microbial communities remains unclear. Therefore, it is crucial to conduct a comparative analysis of the metagenomic sequencing data of the sponge-associated microbiome with previously published metagenomic data from sediment and seawater samples collected at this cold seep [24].

## 2. Materials and Methods

### 2.1. Sample and Data Collections

The F Site cold seep is situated on the northeastern continental slope of the South China Sea. It has a bathymetric range of 1120 to 1155 m and covers an area of roughly 180 m × 180 m, with geographic coordinates ranging from 22.114° N to 22.116° N and 119.285° E to 119.287° E. This study integrates samples collected across different years to facilitate a comprehensive comparison. Specifically, the sponge samples were newly obtained from the site F cold seep during a 2021 expedition [25,26], while the comparative metagenomic data for seawater and sediment were derived from samples collected during the earlier 2017 KEXUE expedition to the same location [24].

In total, this study involved 16 sponge samples from six species, four seawater samples, and sediment samples subdivided into two cores (sediment column 1 and sediment column 2), respectively. For detailed methods regarding sponge sampling, DNA extraction, and identification, please refer to our previously published research [25,26].

Microbial DNA was extracted from sponge samples using the E.Z.N.A.^®^ stool DNA Kit (Omega Bio-tek, Norcross, GA, USA). Due to the lack of a commercially available kit specifically designed for deep-sea sponges, this kit was selected based on its optimal performance in our preliminary DNA extraction experiments. The extraction was conducted following the manufacturer’s protocols. Metagenomic shotgun sequencing libraries were constructed and sequenced at Shanghai Biozeron Biological Technology Co., Ltd. (Shanghai, China). In brief, for each sample, 1 μg of genomic DNA was sheared by Covaris S220 Focused-ultrasonicator (Woburn, MA, USA) and sequencing libraries were prepared with a fragment length of approximately 450 bp.

All samples were sequenced by the Illumina NovaSeq 6000 platform (150bp*2, Shanghai Biozeron Biotechnology Co., Ltd., Shanghai, China). The raw sequencing data have been uploaded to the NCBI Sequence Read Archive (SRA) (http://www.ncbi.nlm.nih.gov/Traces/sra, accessed on 14 November 2024). with the project number PRJNA1186253 specific accession numbers can be found in Table 1. To enhance clarity and convenience in the figures, abbreviations related to sponge taxa will be used to represent sample names. Specifically, “Co,” “Eu,” “Ja,” “Pe,” “Pl,” and “St” denote *Coelosphaera* sp., *Euchelipluma* sp., *Janulum* sp., *Petrosiidae* sp., *Plakina* sp., and *Stylocordyla* sp., respectively. Numbers are used to distinguish between individual samples within the same species.

This dataset includes samples from water closely above the invertebrate communities, cold seep fluids at gas plume, fluid under the invertebrate communities, and sediment columns surrounding reductive sediments area. The metagenomic sequencing data for 23 seawater and sediment samples were acquired from NCBI GenBank (SRA), with accession numbers SRR13892585 to SRR13892607, and the BioProject accession number is PRJNA707313 [24].

### 2.2. Metagenomic Sequence Analyses

The raw sequence reads, which included sponges, seawater, and sediments from the site F cold seep, underwent quality trimming using Trimmomatic v0.36 to remove adaptor contaminants and low-quality reads. Reads through quality control were then mapping against human genome (version: hg38) by BWA mem algorithm. The software parameters are summarized in Supplementary Table S1. The reads removing host-genome contaminations and low-quality data were called clean reads and used for the further analysis. Clean sequence reads were generated to create a set of contigs of for each sample using MegaHit (v1.1.1-2-g02102e1) [27].

The open reading frames (ORFs) of assembled contigs were predicted using MetaProdigal (v2.6.3) [28], and all ORFs were generated to a set of unique genes after clustering using CD-HIT (4.8.1) [29]. The longest sequence of each cluster was selected as the representative sequence of each gene in the unique-gene set. The high-quality reads from each sample were aligned against the unique-gene set using BWA-MEM (v.0.7.17) [30].

Sequence alignment and abundance estimation were performed using Salmon (1.1.0), calculating transcripts per million (TPM). TPM is calculated as follows in equation (1):

(1) $$TPM=\frac{Ng}{Lg}\times \frac{1}{\sum j\frac{Nj}{Lj}}\times 10^{6}$$

*Ng*, the average number of reads mapped to the *g* gene; *Lg*, the number of nucleotides in the *g* gene; The index *j* stands for the set of all genes determined in a catalog, and *g* is an index indicating a particular gene [31].

The unique-gene set was first translated into protein sequences and then searched against the NR database using DIAMOND (0.9.22.123) [32] to identify the gene functions with the following filter parameters. The rarefaction analysis based on Mothur (v.1.21.1) [33] was conducted to reveal the diversity indices by R, including the richness and Shannon diversity indices. We employed a *t*-test to examine the differences between sample groups, and the results of the significance test were presented in a box plot. Utilizing the community composition and abundance data, the Bray–Curtis algorithm was employed to compute pairwise distances between samples. Following this, a hierarchical clustering analysis was conducted based on the distance matrix, utilizing the Unweighted Pair Group Method with Arithmetic Mean (UPGMA) algorithm to construct a hierarchical clustering tree (hclustr tree). After establishing the dendrogram, Hiplot (https://hiplot.com.cn, 2 November 2024) was utilized for visualization and enhancement. Additionally, Non-metric Multidimensional Scaling (NMDS) was applied to represent the Bray–Curtis distances among samples in two-dimensional space, facilitating an understanding of the relationships between groups and samples. Similarity analysis (ANOSIM) was performed to examine the relationship between inter-group differences and intra-group similarities.

### 2.3. Genome Binning and Phylogenomic Analysis

Metagenomic binning was carried out on the contigs of each sample. Initially, the binning software metaBAT2 (v2.11.1) [34], was applied for separate binning processes. The completeness and contamination of all MAGs were assessed using CheckM (v1.1.1) [35]; only high- and medium-quality bins (those with >90% completeness, <5% contamination, and >50% completeness, <10% contamination) were retained for further analyses. The dRep (v3.0.1) [36] tool was employed to remove redundant bins from those classified as medium to high-quality based on an ANI (average nucleotide identity) threshold of >95%. This approach was taken to avoid arbitrary mapping between representatives of highly similar genomes, with the remaining bins designated as MAGs. Taxonomic annotation of all genomes was performed using GTDB-Tk (r226) (2.4.0) [37], a software tool based on the Genome Taxonomy Database (GTDB: http://gtdb.ecogenomic.org/, accessed on 28 April 2024). This tool generated standardized taxonomic labels for subsequent analysis in this study. Genome tree and associated taxonomies were inferred using a set of ubiquitous single-copy proteins. The relative-evolutionary distance (RED) values were then calculated and the results visualized using chiplot (https://www.chiplot.online, accessed on 2 November 2024).

### 2.4. Analysis of Carbon and Sulfur Cycles

The unique-gene set was first translated into protein sequences and then searched against the KEGG database, the carbon cycling database (https://ccycdb.github.io/, accessed on 14 April 2024) and the sulfur cycling database (https://github.com/qichao1984/SCycDB, accessed on 14 April 2024) using DIAMOND (0.9.22.123) [32] for functional annotation. We focused on key metabolic pathways including glycolysis (ko00010), the tricarboxylic acid cycle (ko00020), the pentose phosphate pathway (ko00030), as well as carbon fixation (ko00710, ko00720), sulfur metabolism (ko00920), and methane metabolism (ko00680). Functional analysis was performed using an R package (3.6.3) developed by Shanghai Biozeron Biological Technology Co., Ltd., which enabled the examination of functional gene abundance related to elemental cycling, identification of host microorganisms, and integrated analyses of functional genes, differentially expressed genes, and metabolic pathways. We employed Tukey’s HSD test to analyze inter-group differences, with distinct letters above each group indicating statistically significant differences. Data visualization of the results was performed using the ggplot2 package.

## 3. Results

### 3.1. Community Composition of Site F Cold Seep

**Figure 1:**
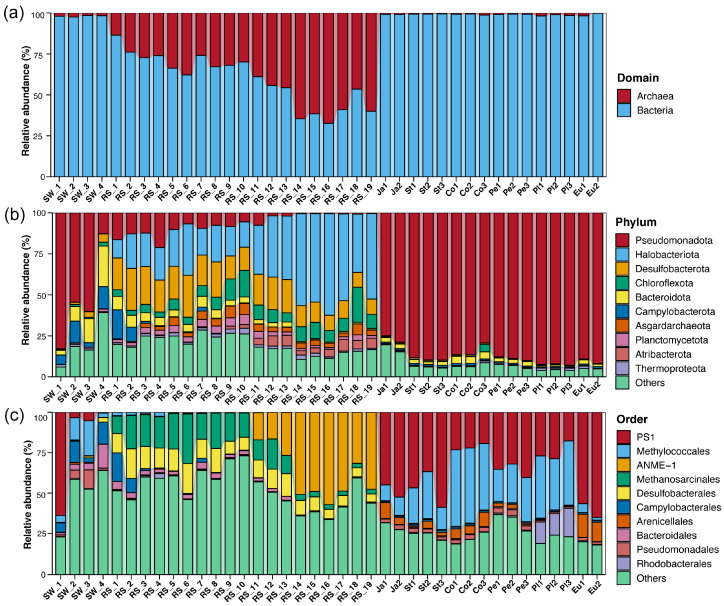
Bacteria and archaea taxonomic composition at (**a**) domain level, (**b**) phylum level, (**c**) order level according to the NR database. Where the x-axis represents the sample names, and the y-axis represents the relative abundance of different microbial taxa in the corresponding samples, respectively. To optimize visual effects, the portions below the top ten were combined into “others” in the bar chart.

It was observed that the relative abundance of archaea was significantly greater than that of bacteria in the sediment samples (Figure 1a), ranging from 13.54% to 67.40% with an average of 40.58%. This characteristic markedly distinguishes them from the archaeal abundances in the seawater microbiome (1.53% to 2.45%) and the sponge-associated microbiome (0.21% to 1.79%). The *t*-test results revealed highly significant differences in archaeal proportions between sediment samples and both the seawater (*p* = 2.9 × 10^−9^) and sponge-associated microbiomes (*p* = 2 × 10^−9^). A significant difference was also observed between the seawater and sponge-associated microbiomes (*p* = 0.0081). Significant differences were observed in archaeal proportions among the sediment, seawater, and sponge-associated microbiomes. The sediment microbiome, in particular, exhibited a significantly higher archaeal proportion. Moreover, a clear trend of increasing archaeal abundance with sediment column depth was identified.

At the phylum level, distinct compositional differences were observed among the sediment, seawater, and sponge-associated microbiomes (Figure 1b). The sponge-associated microbiome was overwhelmingly dominated by Pseudomonadota (average 88.46%), a proportion substantially higher than that in seawater (38.62%). In seawater, Bacteroidota constituted the second most abundant phylum (17.77%). In contrast, the sediment microbiome was characterized by low abundances of both Pseudomonadota and Bacteroidota (each 6.62%), and was instead primarily composed of Halobacteriota (32.51%), Desulfobacterota (17.07%), and Chloroflexota (8.18%).

At the order level, distinct microbial compositions were observed across the sponge, sediment, and seawater microbiomes (Figure 1c). All sponge individuals from the Site F cold seep were characterized by a high prevalence of sulfur-oxidizing bacteria (SOB, order PS1) and methane-oxidizing bacteria (MOB, order Methylococcales). In contrast, the sediment microbiome was dominated by anaerobic methane-oxidizing archaea ANME-1 (Halobacteriota; 18.41%) and methanogenic Methanosarcinales (13.3%), followed by the bacterial order Desulfobacterales (10.99%), reflecting the important role of the anaerobic sediment environment in shaping and sustaining these communities. Conversely, the seawater microbiome lacked distinctly dominant groups, with the most relatively abundant orders being Methylococcales (10.51%), Campylobacterales (9.46%), Bacteroidales (8.83%), and Flavobacteriales (7.17%).

### 3.2. α-Diversity Analysis

**Figure 2:**
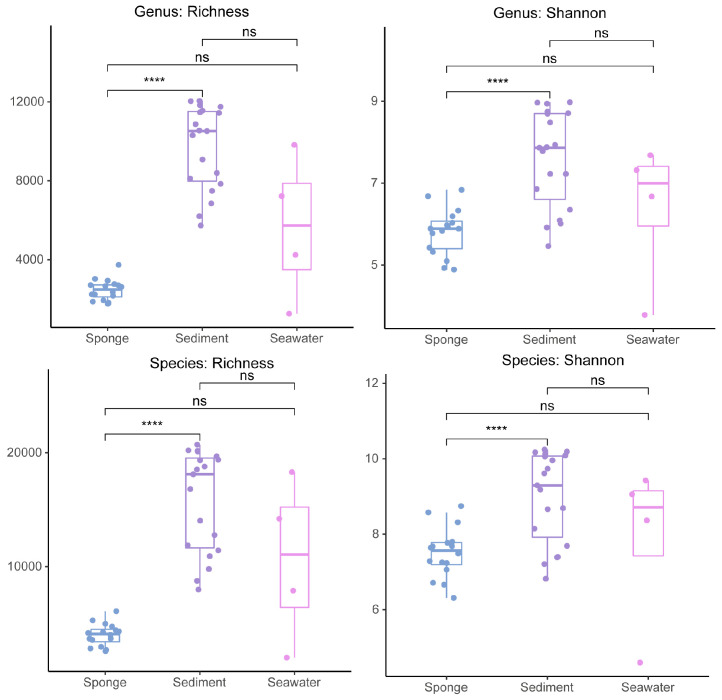
Boxplot of α-diversity for three source samples from the site F cold seep. Box plots depict the distribution of data across sample groups. Statistical significance between groups was assessed using a *t*-test and is indicated above the plots: ****, *p* < 0.00001; ns., not significant.

α-Diversity refers to the richness of species or functions within a specific habitat or ecosystem and can indicate the balance state and conditions for survival in that habitat. A comparative analysis of the α-diversity among the sediment, seawater microbiome, and sponge-associated microbiome at site F cold seep was conducted. We calculated alpha diversity at both the genus and species levels. Richness and Shannon indices were calculated and visualized using box plots (Figure 2).

After analyzing the α-diversity metrics of sponges, sediments, and seawater, significant differences among these sources can be observed. In terms of richness, sediments exhibited generally high values ranging from 6202 to 11,836. The richness of seawater samples varied considerably, ranging from 1261 to 9821, with individual samples (e.g., SW_4: fluid under the invertebrate communities) showing particularly high richness. Conversely, the richness of sponges was relatively low, ranging from 1760 to 3737, indicating fewer species types. Difference tests reveal that the species richness in sediments is significantly higher than that in sponges and seawater, suggesting greater microbial species diversity in sediments. Sediments have the highest richness, sponges the lowest, and seawater is highly variable.

Regarding the Shannon index, sediments displayed the highest Shannon index, indicating a more even distribution of abundance among species in its ecosystem and richer species diversity. Some seawater samples (e.g., SW_4) also exhibited high diversity, although overall, they remained lower than sediments. The Shannon index for sponges was the lowest, reflecting poor evenness among species, suggesting dominance by certain species while others are relatively sparse. The results of the difference tests show that the Shannon index for sediments is significantly higher than that for seawater and sponges, indicating a more uniform species distribution in sediments.

### 3.3. β-Diversity Analysis

**Figure 3:**
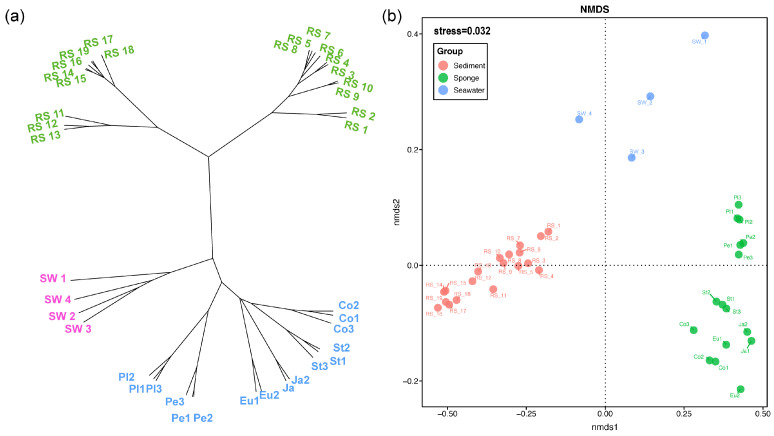
β-diversity for three source samples from the site F cold seep. (**a**) Hcluster Tree Based on Bray–Curtis Distance; (**b**) Non-metric Multidimensional Scaling (NMDS) Based on Bray–Curtis Distance.

The hierarchical clustering tree (Hcluster) provides a clearer representation of the differences among various samples (Figure 3a). The tree can be primarily divided into four branches, representing: sponges, seawater, sediment column 1, and sediment column 2. Within the sponge branch, samples of the same sponge species are located closer together, whereas different sponge species are more distantly spaced. In the sediment branch, distances are observed between different sediment columns; specifically, within sediment column 1, there are further subdivisions based on depth: 0–4 cm below seafloor (cmbsf), 4–16 cmbsf, and 18–20 cmbsf. In sediment column 2, there are two smaller groups: 0–90 cmbsf and 90–900 cmbsf. This indicates that the sampling locations and depths of the sediment columns have a significant impact on community composition, illustrating both horizontal and vertical variability within the sediment microbiome.

Non-metric Multidimensional Scaling (NMDS) results (Figure 3b) were consistent with the Hcluster tree, showing clear separation among the three groups—seawater, sediment microbiome, and sponge-associated microbiome—with inter-group differences substantially greater than intra-group variations. This highlights the distinct microbial diversity of different sources at site F cold seep. Intra-group differences were influenced by factors such as sponge species, sediment depth, and sampling location. The NMDS analysis showed a stress value of 0.032 (<0.2), indicating a reliable ordination fit. ANOSIM further confirmed significant community segregation, with an R value of 0.99 (*p* = 0.001), demonstrating that between-group similarities are significantly lower than within-group similarities, and thus reflecting substantial differences in microbial composition among sediments, seawater, and sponge-associated microbiomes.

### 3.4. Archaeal Communities Based on MAGs

**Figure 4:**
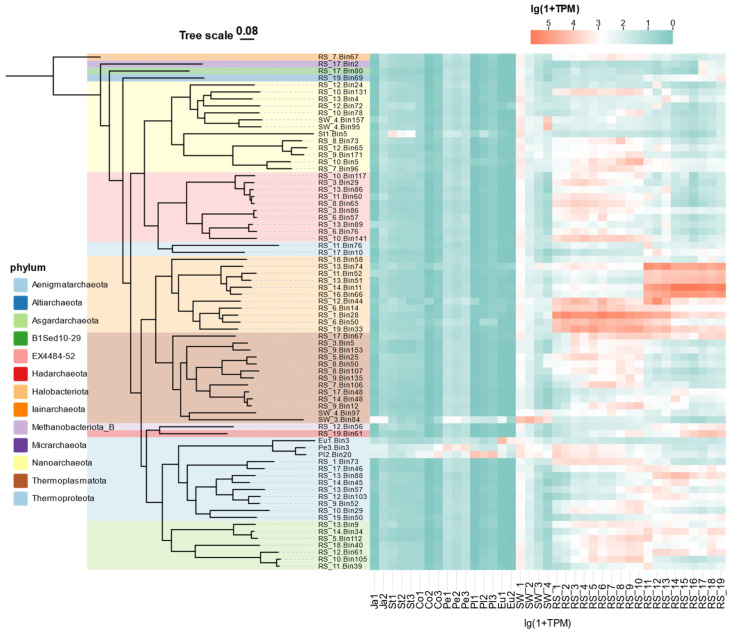
An archaeal genome tree and associated taxonomies inferred using a set of 53 ubiquitous single-copy proteins and relative-evolutionary distance (RED) values. A TPM-based heatmap was generated to visualize the relative abundance differences between the different MAGs adjacent to the phylogenetic tree. Detailed classification of all archaeal MAGs and additional information can be found in Table S2.

From the metagenomic sequencing results of seawater, sediment, and sponges at site F cold seep, a total of 74 medium-to-high quality archaeal MAGs were recovered. These archaeal MAGs were constructed a phylogenetic tree using GTDBtk. A heatmap was generated based on TPM to visualize the relative abundance differences among the various MAGs (Figure 4, Table S2).

The phylogenetic status represented by the archaeal MAGs and their abundance information reveal differences between the microbial communities in seawater, sediment, and sponge associations at site F cold seep. The expression levels of archaea within the sponge microbiome are relatively low, primarily concentrated in the phylum Thermoproteota. In contrast, the sediment exhibits significantly higher expression levels of archaea from the dominant phylum Halobacteriota, with different genotypes related to Halobacteriota from the two sediment columns indicating phylogenetic variability. Additionally, groups from the Asgardarchaeota, EX4484-52, and Altiarchaeota phyla also show relatively high expression levels. It can be observed that the expression levels of most archaea in sediments are higher than those in sponges and seawater, which may be related to the anaerobic adaptability of archaea.

### 3.5. Analysis of Bacterial Communities Based on MAGs

**Figure 5:**
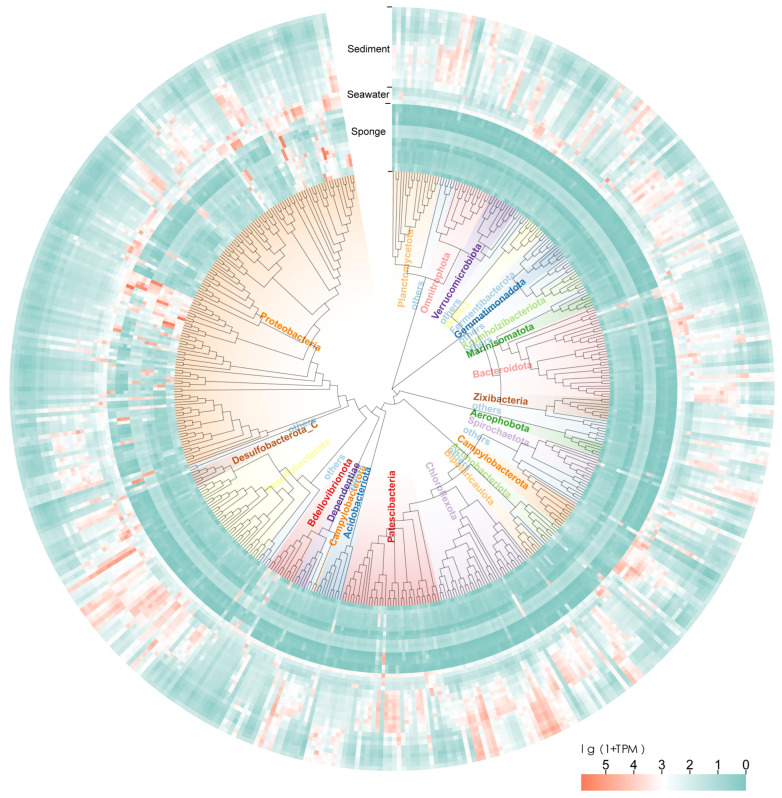
A bacterial genome tree and associated taxonomies inferred using a set of 120 ubiquitous single-copy proteins and relative-evolutionary distance (RED) values. The adjacent TPM-based heatmap visualizes the relative abundance differences in these MAGs across sample types. Detailed classification of all bacterial MAGs and additional information can be found in Table S3.

From the metagenomic sequencing results of seawater, sediment, and sponges at site F cold seep, a total of 409 medium-to-high quality bacterial MAGs were recovered. These archaeal MAGs were constructed a phylogenetic tree using GTDBtk. A heatmap was generated based on TPM to visualize the relative abundance differences among the various MAGs (Figure 5, Table S3).

The phylogenetic status represented by the bacterial MAGs and their abundance information reveal differences between the microbial communities in seawater, sediment, and sponge associations at site F cold seep. From the perspective of MAG abundance, it is evident that the dominant SOB (order PS1) and MOB (order Methylococcales) related MAGs present within the sponge do not exhibit dominant expression in the background seawater and sediment. Similarly, individuals of MAGs that exhibit relatively high abundance in seawater and sediments are present at lower levels within the sponges. Although sponges filter seawater through aquifer systems and connect to the seafloor at their base, the dominant microbial genotypes within them differ from those in the seawater and sediment. The differences between the sponge-associated microbiome and the seawater and sediment microbiomes may be related to the adaptability of the sponge-microbe symbiosis to a symbiotic lifestyle.

### 3.6. Genome Size and GC Content

**Figure 6:**
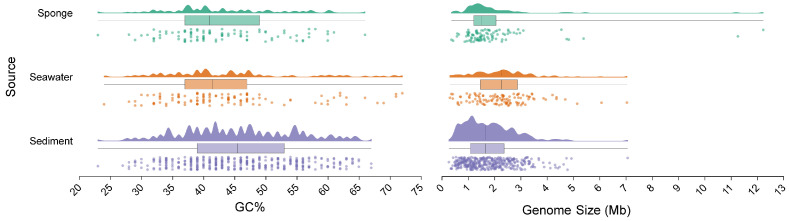
The distribution map of genome size and GC content based on MAGs.

When studying microbial ecology and their interactions with hosts or the environment, genomic size and GC content are important reference indicators. In this study, MAGs of seawater, sediment, and sponge were used to create raincloud plots and box plots depicting genome size and GC content (Figure 6). The results indicate that, compared to seawater, the MAGs from sponge-associated microbial communities and sediments are smaller, with the genomic size of sponge-associated MAGs being slightly larger than that of sediment MAGs. Furthermore, the *t*-test results showed that the GC content of the sponge-associated microbial community was significantly lower than that of the sediment and seawater communities.

As noted previously, among the microorganisms within the sponge, chemolithotrophic microbes exhibit the highest relative abundance, dominating the community. The genomic sizes of these relevant chemolithotrophic microbes were further investigated. Generally, the genomes of chemolithoautotrophic symbionts range from 1.02 to 4.88 MB, with variability influenced by metabolic strategies [38]. In this study, the average genomic sizes for sponge-associated PS1 and Methylococcales were found to be 1.12 MB and 1.88 MB, respectively, placing them within the lower range of chemolithoautotrophic symbionts. This characteristic reflects adaptations to a symbiotic lifestyle and extreme environments found in deep-sea cold seeps. By retaining complete sulfur and methane metabolism functions while reducing other metabolic coding and energy expenditure, this trait aligns with the general characteristics of vertical transmission and a high degree of symbiotic cooperation with the host.

### 3.7. Differential Analysis of Carbon and Sulfur Metabolic Genes

**Figure 7:**
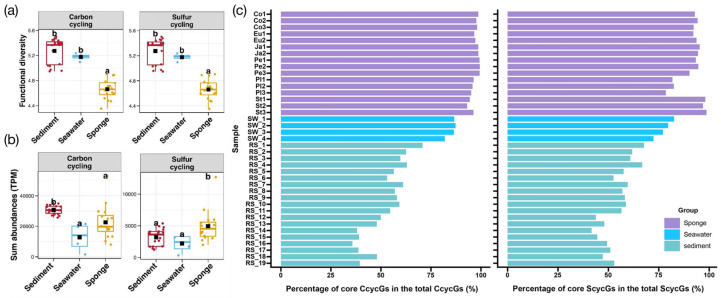
Differential Analysis of Carbon and Sulfur Metabolic Genes (**a**) Comparison of differences in the diversity composition of functional genes involved in carbon and sulfur cycling. The presence of different letters between any two groups denotes a significant difference at a predetermined significance level (typically *p* < 0.05); (**b**) comparison of differences in the overall compositional structure of functional genes related to carbon and sulfur cycling; (**c**) percentage of core genes in carbon and sulfur cycling genes among different samples.

Differential analysis was conducted on the total number of detected subtypes of carbon and sulfur cycling functional genes from samples collected from sediment, seawater, and sponges to compare the diversity of elemental cycling functions among different groups (indicated by the Shannon index, Figure 7a). A significance test for the total abundance of carbon and sulfur cycling functional genes in each group was also performed to compare inter-group differences in total gene abundances. The results are presented using scatter jitter plots (Figure 7b).

The results indicate that sponge-associated microbial communities exhibit significantly lower gene diversity related to carbon and sulfur cycling functional genes compared to environmental microbial communities (sediment and seawater). However, in terms of total carbon cycling gene abundance, sediment exhibited significantly higher gene abundance than sponge-associated microbial communities and seawater. Conversely, sponge-associated microbes displayed significantly greater total sulfur cycling gene abundance than those found in seawater and sediment. Overall, sponge-associated microbial communities demonstrate lower gene diversity in carbon and sulfur cycling compared to sediment and seawater microbial communities but express higher levels of sulfur cycling genes and relatively high levels of carbon cycling genes. This suggests that sponge-associated microbial communities concentrate most carbon and sulfur cycling genes within a limited number of microbial taxa, which may be crucial for the symbiotic lifestyle of these microbes within sponges. In contrast, sediment and seawater possess higher diversity in carbon and sulfur cycling genes, facilitating more complex metabolic functions.

The degree of diversity concerning carbon and sulfur cycling-related genes in sponge-associated microbes is further reflected in the proportion of core genes. In this study, we define the core gene set of carbon/sulfur cycling functional genes (based on the KO numbers from the KEGG database) as those that are detected in all individual samples analyzed. Based on the identified core carbon cycling functional genes, their total abundance in each sample was calculated to determine the proportion of these core genes relative to the total genome in each sample, allowing for assessment of the contribution of the core gene set to the overall carbon and sulfur cycling functional gene pool. Variations in these proportions across different samples were illustrated in bar graph format (Figure 7c).

The proportion of core carbon cycling genes in sponges reached between 93.06% and 99.42% (these percentages are relative to the total genes for that cycle in each sample, hereinafter the same), while core sulfur cycling genes constituted between 78.49% and 98.66%, both of which were higher than those found in environmental sediments and seawater. This indicates a relatively low specificity of carbon cycling genes present in sponge-associated symbiotic microbes, suggesting that these microbes only need to express basic carbon cycling functions to meet their essential survival needs. On one hand, this points to a survival strategy in sponge-associated microbes that favors smaller genomes, discarding non-essential components that could be compensated for by environmental microbes. On the other hand, it implies a potential cooperative relationship regarding carbon cycling functions between sponge-associated microbes and their host, although the technical challenges involved in sequencing the sponge genome currently hinder verification of this relationship.

### 3.8. The Carbon and Sulfur Element Cycling Model

**Figure 8:**
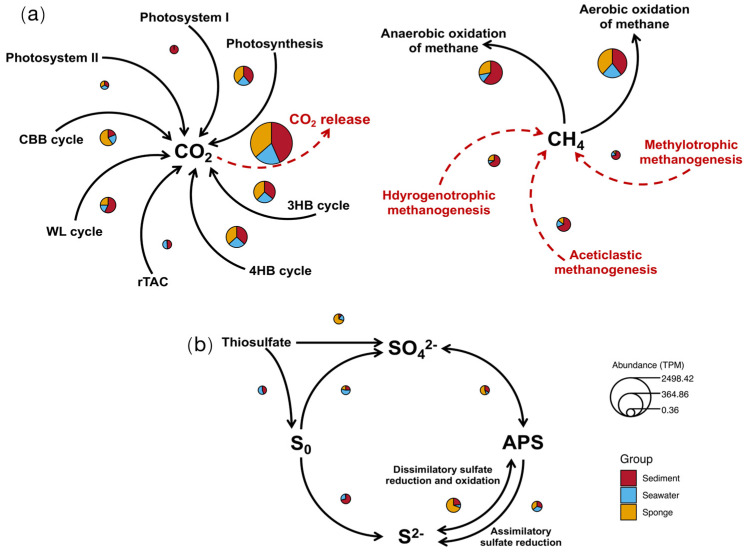
The carbon and sulfur element cycling model. (**a**) The carbon element cycling model diagram of the site F cold seep; (**b**) the sulfur element cycling model diagram of the Site F cold seep.

The carbon cycling process primarily includes the fixation and release of CO_2_, as well as the fixation and release of CH_4_. In specific elemental cycling pathways (Figure 8a), sponge-associated microbial communities encode a higher abundance of genes related to the Calvin-Benson-Bassham (CBB) cycle compared to environmental seawater and sediment microbial groups. The CBB cycle is capable of effectively fixing more carbon with lower energy input, while its reaction products can be recycled, thereby reducing energy and material waste; it represents a relatively energy-efficient method for carbon fixation. In contrast, the sediment and seawater predominantly employ the reversed citric acid (rTCA) cycle and the reductive acetyl-CoA pathway for carbon fixation.

Additionally, both environmental and sponge microbial communities exhibit high abundances of genes associated with the 3-hydroxypropionate cycle and the 4-hydroxybutyrate cycle. These cycles are linked to energy storage and release, the degradation and recycling of organic matter, participation in nitrogen cycling, and the production of biopolymers, all of which provide significant support for the flow of materials and energy within the ecosystem. The high abundance of genes encoding CO_2_ release indicates the vigorous metabolic potential at site F cold seep, particularly within the sponge-associated microbial community.

Due to the anoxic environment characteristic of sediments, the abundance of anaerobic methane oxidation pathways is significantly higher than that found in seawater and sponge-associated microbial communities. Additionally, the abundance of methane-generating genes in sediments is also markedly higher than in seawater and sponge-associated microbes, likely related to microbial anaerobic metabolism and substrate utilization under anoxic conditions.

The inorganic sulfur cycling process exhibits differences in abundance across microbial communities from various sources (Figure 8b). During the inorganic sulfur cycle, sponge-associated microbial communities encode a higher abundance of genes involved in the redox processes of thiosulfate, sulfate, adenosine phosphosulfate (APS), and sulfide (S^2−^), whereas genes related to the reduction in thiosulfate and elemental sulfur are predominantly encoded in seawater and sediment environments. Specifically, sponge-associated microbial communities show a higher abundance of genes encoding sulfate-reducing enzyme (*apr*), dissimilatory sulfite reductase (*dsr*), and methionine synthase (*metZ*), each playing different roles in sulfur metabolism while collectively promoting the transformation and utilization of sulfur. For instance, the sulfite produced by the *apr*-catalyzed reaction can be further processed by *dsr*, while *metZ* utilizes these sulfur sources to synthesize amino acids. The high abundance of these genes relative to the environment may be closely related to the nutrition and energy needs of the symbiotic organisms.

## 4. Discussion

### 4.1. Differences in Community Composition Between the Sponge-Associated Microbiome and Environmental Microbiome at Site F Cold Seep

Through comparative metagenomic sequencing analysis of sediments, seawater microbiomes, and sponge-associated microbiomes from Site F cold seep, We demonstrated distinct differences in community composition between sponge-associated microbiomes and environmental microbiomes. These differences are manifested in the structure of community composition, α-diversity, β-diversity, and the genomic characteristics of MAGs.

The sponge-associated microbiome is predominantly composed of bacteria, with Proteobacteria being the absolute dominant phylum. Notably, members of the order Methylococcales and order PS1, are significantly enriched in the sponge microbiome compared to the communities found in seawater and sediments. A key feature of sponge-associated microbial communities is the enrichment of specific bacterial groups termed “sponge-specific clusters”, which are often low or absent in surrounding waters [23,39,40]. In this study, we propose that the differential abundance and genotype expression of order PS1 and Methylococcales members between sponges and their environment align with the hypothesis of “sponge-specific clusters”. In contrast, archaeal groups that are abundant in sediments show very low abundance in sponges.

Additionally, the significantly lower α-diversity observed in the sponge-associated microbiome, with reduced richness at both the species and genus levels, likely reflects a specialized symbiotic adaptation. This streamlined community suggests a selective process for highly efficient microbial partners, rather than a stochastic assemblage. β-diversity analysis indicates that inter-group differences between sponge-associated and environmental microbiomes exceed intra-group variability. Although there is host-specificity in the community composition of sponge-associated microbiomes at the host species level, the intra-sponge sample correlation remains stronger than comparisons with seawater and sediment samples, suggesting that the sponge-associated microbiome represents a specialized and adaptive community associated with the sponge host.

Metagenomic assembly revealed that evolutionary and abundance distinctions between the sediment and seawater microbiomes, as compared to the sponge-associated microbiome, extend to the single-genus level. Critically, at the resolution of metagenome-assembled genomes (MAGs), our analysis demonstrates a clear genetic distance between dominant sponge-associated genotypes and their environmental counterparts—even among organisms of the same phylum at Site F cold seep. This genotypic resolution allows discrimination of host-specific microbial populations that would likely remain indistinguishable using less detailed, non-genomic methods. Furthermore, the sponge-associated microbiome was characterized by smaller genome sizes and lower GC content relative to environmental microbiomes. This genomic reduction implies a lower metabolic cost for replication and maintenance, representing a resource conservation strategy typical of host-dependent symbionts and reinforcing its distinct evolutionary trajectory.

This study highlights the various differences between sponge-associated microbial communities and environmental microbial communities, which may have important implications for sponge symbiosis. Compared to other ecosystems, chemoautotrophic ecosystems present a relatively harsh ecological environment due to factors such as low temperature, high pressure, and limited nutrients. Although sponges are the most primitive multicellular animals with a simple structure, from the perspective of community composition, the microbial community forms a close and specific mutualistic relationship with the “simple multicellular animals” of sponges, resulting in a microbial community that is markedly different from the surrounding environment. Additionally, the observed variability within sampling groups suggests that microenvironmental differences contribute to microbial community disparities in the Site F cold seep region.

However, the sponge samples used in this study were collected in 2021, while the environmental samples were obtained in 2017. Although our comparison between the sponge-associated microbiome and the environmental microbiome at Site F cold seep revealed relevant differences in community composition, we must acknowledge that microbial communities in deep-sea environments are sensitive to seasonal, interannual, or event-driven fluctuations in geochemical conditions—such as methane flux, sulfide concentration, or oxygen availability. The inconsistency in sampling timing may thus affect the comparability of the data. In subsequent experimental designs, we will place greater emphasis on covering all necessary sample types within a single sampling expedition as comprehensively as possible.

### 4.2. Functional Coding Differences Between the Sponge-Associated Microbiome and Environmental Microbiome in Site F Cold Seep

Site F cold seep is a typical representative of chemolithoautotrophic environments, where microbial communities are involved in extensive chemolithoautotrophic reactions. This study discusses the relevant functional genes related to carbon and sulfur cycling in sediments, seawater microbiomes, and sponge-associated microbiomes at Site F cold seep. It has been found that there are differences in the functional gene coding between the sponge-associated microbiome and environmental microbiomes. Compared to sediment and seawater microbiomes, the sponge-associated microbiome exhibits significantly lower genetic diversity.

The genetic repertoire for carbon and sulfur cycling in the sponge-associated microbiome is highly streamlined, retaining only the core set of genes essential for community function. Rather than maintaining a broad genetic “catalog” as seen in sediments, the sponge microbiome operates with a compact, specialized, and highly efficient “toolkit,” precisely tailored to the host’s metabolic requirements. Functionally, the abundance of carbon cycling genes in the sponge microbiome was comparable to that in seawater but significantly lower than in sediments. In contrast, sulfur cycling genes were markedly enriched in sponges relative to both sediment and seawater. This prominent encoding of sulfur metabolism genes further underscores their critical role in sponge symbiosis.

Differential expression analysis of metabolic pathways revealed that sponge-associated microorganisms exhibit significantly higher gene abundances in key processes including the Calvin-Benson-Bassham (CBB) cycle, thiosulfate oxidation, sulfate reduction, and dissimilatory sulfate reduction. The prominent expression of the CBB cycle indicates a strong capacity for autotrophic carbon fixation, establishing these symbionts as primary nutrient providers for the host sponge in the chemosynthetically driven cold seep environment. These genomic observations would suggest that sponge-microbe symbionts could utilize specific chemical substances in the environment (such as sulfides) and potentially complete material transformation and energy acquisition through diverse metabolic pathways. This further implies that the sponge-associated microbiome might occupy a distinct ecological niche and exhibit specific adaptations in energy acquisition and nutrient cycling compared to environmental microbiomes. Moreover, these findings would point to the potential contributions of sponge-microbe symbionts to carbon and sulfur cycling in the Site F cold seep.

Anaerobic methane oxidation (AOM) is an important biogeochemical process in deep-sea cold seeps, capable of consuming approximately 90% of the methane in sediments. This process plays a crucial role in regulating global methane levels and mitigating the greenhouse effects caused by methane [41]. AOM is mediated by anaerobic methanotrophic archaea (ANME) and is usually coupled with the sulfate-reducing processes driven by sulfate-reducing bacteria (SRB), referred to as sulfate-dependent anaerobic methane oxidation (SAMO) [42,43]. The high abundance coding of ammonia-oxidizing archaea and genes associated with ammonia oxidation and methanogenesis in Site F cold seep sediments implies their significant role in the methane material cycling at Site F. Given the close association of microorganisms living in cold seeps with methane production and conversion, as well as the potential for natural gas hydrate resource exploitation in cold seep areas, enhancing research on functionally relevant microorganisms related to methane metabolism in Site F cold seep sediments will be an important direction for advancing our understanding and development of cold seep ecosystems.

### 4.3. Symbiotic Adaptations of Sponge-Associated Microbiomes at Site F Cold Seep

Through metagenomic sequencing results of environmental microbes from sediment and seawater at Site F cold seep, this study observed the symbiotic adaptations of sponge-associated microbiomes. Compared to the environmental background, the sponge-associated microbiomes exhibited lower diversity and simpler community composition, encoding less complex carbon and sulfur cycling functional genes while performing metabolic functions essential for the survival of the symbionts. Overall, the response mechanisms of sponge-associated microbiomes to their symbiotic lifestyle include: dominant taxa occupying high abundance within the community; smaller genome sizes and lower GC content; as well as differences in carbon and sulfur cycling gene types and abundances.

Changes in genome size and GC content are often used to reflect microbial adaptation and evolutionary processes in specific environments. These changes are typically associated with factors such as genome simplification and adaptation, DNA stability, and selective pressures [44]. Genome reduction is closely linked to microbial lifestyle; parasitic or symbiotic microbes often harbor smaller genomes as they rely on their hosts for nutrients and environmental stability [38]. The Black Queen Hypothesis (BQH) provides a compelling framework for interpreting this pattern in our study: symbionts within sponges lose non-essential genes while retaining those critical for biomass synthesis and core metabolism, thereby enhancing growth efficiency and minimizing metabolic costs [45]. This genomic streamlining is facilitated by the stable, nutrient-rich environment provided by the host sponge, which compensates for the loss of certain metabolic functions. In extreme free-living environments (e.g., high temperature or acidity), elevated GC content can help maintain DNA stability [46]. The observed lower GC content in sponge-associated microbes may thus reflect the buffered and secure nature of their host-derived niche. While the observed patterns are consistent with the phenomenon of genome streamlining in symbionts, this study did not perform a detailed analysis of genomic features such as pseudogene accumulation, specific auxotrophies, or the loss of biosynthetic pathways that would provide direct evidence for reductive evolution and functional dependency. Future work will involve a dedicated comparative genomic analysis of these high-quality MAGs, focusing on identifying these specific hallmarks to conclusively elucidate the mechanisms and evolutionary trajectory of symbiosis in this unique cold-seep ecosystem.

Interestingly, previous studies have found that the metagenome of sponges exhibits higher GC content and larger genome sizes compared to that of seawater, with the increased genome size attributed to higher horizontal gene transfer levels within the sponge hosts compared to the marine environment [47]. The hypothesis of increased gene transfer levels is supported by the discovery of numerous mobile genetic elements and transposases required for genetic transfer in the genomic library of sponge symbionts; these factors are likely important for the evolutionary adaptation of sponge microbiomes to a symbiotic lifestyle [48,49]. This characteristic parallels the findings in this chapter, where sponge-associated microbiomes exhibit relatively higher GC content and larger genome sizes compared to sediments, although it remains undetermined whether this is related to the presence of genetic elements, necessitating further investigation.

Based on prior research, this study posits that the “sheltering” and “cooperative” nature of the symbiotic relationship with host sponges mitigates environmental stress on microbial communities, compensating for the reduced resistance stability of communities due to a lack of diversity. Consequently, chemoautotrophic functional groups, which have significant cooperative relationships with sponges, become dominant taxa within these communities, establishing specific and enriched symbiotic relationships. The characteristics of smaller genome sizes and lower GC content, along with potential vertical transmission pathways, provide crucial foundational conditions for the prevalence of functional groups exclusively within sponge populations.

## 5. Conclusions

In this study, we utilized metagenomic datasets from Site F cold seep to conduct a comparative metagenomic analysis of sponge-associated microbiomes, seawater, and sediment microbial communities. The results revealed significant inter-group differences in community structure, diversity, and functional gene encoding, which not only delineate clear boundaries between symbiotic and free-living lifestyles but also highlight the distinct adaptations of microbial communities to these niches at Site F cold seep. We validated that the dominant functional microbial groups are, in fact, sponge-specific clusters, providing definitive genomic evidence for the existence of host-adapted lineages within the cold seep ecosystem. Regarding carbon and sulfur metabolism, the sponge-associated microbiome exhibited a “specialized and efficient” coding strategy for carbon and sulfur genes, which may better align with the energy requirements of the sponge-microbe symbiosis. This research focused on the sponge-associated microbiomes and environmental microbial communities at Site F cold seep, revealing their characteristics in community composition, functional encoding, symbiotic adaptation, and environmental adaptation. It provides new data support and insights for understanding the composition and functionality of microbes in this unique habitat. Advancements in technology can further elucidate how symbiotic organisms acquire energy from their environment and provide metabolites and nutrients to their hosts. Additionally, future studies should aim to clarify the functional roles of different symbiotic microbial taxa. Quantitative measurements of metabolite fluxes (e.g., methane and sulfides) are also needed to provide direct evidence for the metabolic functions inferred from metagenomic data, which is crucial for a mechanistic understanding of cold seep and hydrothermal vent ecosystems.

## Figures and Tables

**Table 1:** The abbreviation status of sponge samples and the NCBI accession numbers.

Sample Name	Abbreviation	Biosample_Accession	SRR_Accession
*Coelosphaera* sp._1	Co1	SAMN44752447	SRR31358909
*Coelosphaera* sp._2	Co2	SAMN44752448	SRR31358908
*Coelosphaera* sp._3	Co3	SAMN44752449	SRR31358907
*Euchelipluma* sp._1	Eu1	SAMN44752456	SRR31358914
*Euchelipluma* sp._2	Eu1	SAMN44752457	SRR31358913
*Janulum* sp._1	Ja1	SAMN44752458	SRR31358912
*Janulum* sp._2	Ja2	SAMN44752459	SRR31358911
*Petrosiidae* sp._1	Pe1	SAMN44752453	SRR31358903
*Petrosiidae* sp._2	Pe2	SAMN44752454	SRR31358916
*Petrosiidae* sp._3	Pe3	SAMN44752455	SRR31358915
*Plakina* sp._1	Pl1	SAMN44752450	SRR31358906
*Plakina* sp._2	Pl2	SAMN44752451	SRR31358905
*Plakina* sp._2	Pl2	SAMN44752452	SRR31358904
*Stylocordyla* sp._1	St1	SAMN44752444	SRR31358918
*Stylocordyla* sp._2	St2	SAMN44752445	SRR31358917
*Stylocordyla* sp._3	St3	SAMN44752446	SRR31358910

## Data Availability

The original data presented in the study are openly available in GenBank at PRJNA1186253.

## Supplementary Materials

The following supporting information can be downloaded at: https://www.mdpi.com/article/10.3390/microorganisms13122672/s1, Table S1: The parameters and version of the main software described in the Section 2; Table S2: Information for the 74 archaeal MAGs; Table S3: Information for the 501 bacterial MAGs.

## References

[1] Olu K., Duperret A., Sibuet M., Foucher J., Fiala-Médioni A. (1996). Structure and distribution of cold seep communities along the Peruvian active margin:relationship to geological and fluid patterns. Mar. Ecol. Prog. Ser..

[2] Feng D., Qiu J.-W., Hu Y., Peckmann J., Guan H., Tong H., Chen C., Chen J., Gong S., Li N. (2018). Cold seep systems in the South China Sea: An overview. J. Asian Earth Sci..

[3] Feng D., Cheng M., Kiel S., Qiu J.-W., Yang Q., Zhou H., Peng Y., Chen D. (2015). Using Bathymodiolus tissue stable carbon, nitrogen and sulfur isotopes to infer biogeochemical process at a cold seep in the South China Sea. Deep. Sea Res. Part I Oceanogr. Res. Pap..

[4] Zhang X., Du Z., Luan Z., Wang X., Xi S., Wang B., Li L., Lian C., Yan J. (2017). In Situ Raman Detection of Gas Hydrates Exposed on the Seafloor of the South China Sea. Geochem. Geophys. Geosyst..

[5] Liang Q., Hu Y., Feng D., Peckmann J., Chen L., Yang S., Liang J., Tao J., Chen D. (2017). Authigenic carbonates from newly discovered active cold seeps on the northwestern slope of the South China Sea: Constraints on fluid sources, formation environments, and seepage dynamics. Deep. Sea Res. Part I Oceanogr. Res. Pap..

[6] Cao L., Lian C., Zhang X., Zhang H., Wang H., Zhou L., Wang M., Chen H., Luan Z., Li C. (2021). In situ detection of the fine scale heterogeneity of active cold seep environment of the Formosa Ridge, the South China Sea. J. Mar. Syst..

[7] Gong L., Li X., Qiu J.-W. (2015). Two new species of Hexactinellida (Porifera) from the South China Sea. Zootaxa.

[8] Levin L.A., Baco A.R., Bowden D.A., Colaco A., Cordes E.E., Cunha M.R., Demopoulos A.W.J., Gobin J., Grupe B.M., Le J. (2016). Hydrothermal Vents and Methane Seeps: Rethinking the Sphere of Influence. Front. Mar. Sci..

[9] Kochevar R.E., Childress J.J., Fisher C.R., Minnich E. (1992). The methane mussel: Roles of symbiont and host in the metabolic utilization of methane. Mar. Biol..

[10] Li X. (2017). Taxonomic research on deep-sea macrofauna in the South China Sea using the Chinese deep-sea submersible *Jiaolong*. Integr. Zool..

[11] Sogin E.M., Kleiner M., Borowski C., Gruber-Vodicka H.R., Dubilier N. (2021). Life in the Dark: Phylogenetic and Physiological Diversity of Chemosynthetic Symbioses. Annu. Rev. Microbiol..

[12] Goffredi S.K., Tilic E., Mullin S.W., Dawson K.S., Keller A., Lee R.W., Wu F., Levin L.A., Rouse G.W., Cordes E.E. (2020). Methanotrophic bacterial symbionts fuel dense populations of deep-sea feather duster worms (Sabellida, Annelida) and extend the spatial influence of methane seepage. Sci. Adv..

[13] Wang B., Du Z., Luan Z., Zhang X., Wang M., Wang X., Lian C., Yan J. (2021). Seabed features associated with cold seep activity at the Formosa Ridge, South China Sea: Integrated application of high-resolution acoustic data and photomosaic images. Deep. Sea Res. Part I Oceanogr. Res. Pap..

[14] Tokuda G., Yamada A., Nakano K., Arita N.O., Yamasaki H. (2010). Colonization of Sulfurovum sp. on the gill surfaces of Alvinocaris longirostris, a deep-sea hydrothermal vent shrimp. Mar. Ecol..

[15] Qing-Lei S., Zhi-Gang Z., Shuai C., Li S., Kellogg C.A. (2016). First Comparative Analysis of the Community Structures and Carbon Metabolic Pathways of the Bacteria Associated with Alvinocaris longirostris in a Hydrothermal Vent of Okinawa Trough. Plos One.

[16] Zhou K., Zhang R., Sun J., Zhang W., Tian R.-M., Chen C., Kawagucci S., Xu Y. (2019). Potential Interactions between Clade SUP05 Sulfur-Oxidizing Bacteria and Phages in Hydrothermal Vent Sponges. Appl. Environ. Microbiol..

[17] Georgieva M.N., Taboada S., Riesgo A., Díez-Vives C., De Leo F.C., Jeffreys R.M., Copley J.T., Little C.T.S., Ríos P., Cristobo J. (2020). Evidence of Vent-Adaptation in Sponges Living at the Periphery of Hydrothermal Vent Environments: Ecological and Evolutionary Implications. Front. Microbiol..

[18] Rubin-Blum M., Antony C.P., Sayavedra L., Martínez-Pérez C., Birgel D., Peckmann J., Wu Y.-C., Cardenas P., MacDonald I., Marcon Y. (2019). Fueled by methane: Deep-sea sponges from asphalt seeps gain their nutrition from methane-oxidizing symbionts. ISME J..

[19] Xin Y., Wu N., Sun Z., Wang H., Chen Y., Xu C., Geng W., Cao H., Zhang X., Zhai B. (2022). Methane seepage intensity distinguish microbial communities in sediments at the Mid-Okinawa Trough. Sci. Total Environ..

[20] Margalef R. (1963). On Certain Unifying Principles in Ecology. Am. Nat..

[21] Moitinho-Silva L., Nielsen S., Amir A., Gonzalez A., Ackermann G.L., Cerrano C., Astudillo-Garcia C., Easson C., Sipkema D., Liu F. (2017). The sponge microbiome project. GigaScience.

[22] Pita L., Rix L., Slaby B.M., Franke A., Hentschel U. (2018). The sponge holobiont in a changing ocean: From microbes to ecosystems. Microbiome.

[23] Hentschel U., Piel J., Degnan S.M., Taylor M.W. (2012). Genomic insights into the marine sponge microbiome. Nat. Rev. Microbiol..

[24] Zhang H., Wang M., Wang H., Chen H., Cao L., Zhong Z., Lian C., Zhou L., Li C. (2022). Metagenome sequencing and 768 microbial genomes from cold seep in South China Sea. Sci. Data.

[25] Wang Y., Gong L., Gao Z., Wang Y., Zhao F., Fu L., Li X. (2023). Host-specific bacterial communities associated with six cold-seep sponge species in the South China Sea. Front. Mar. Sci..

[26] Wang Y., Gong L., Dong D., Li X. (2025). Metagenomic binning reveals community and functional characteristics of sulfur- and methane-oxidizing bacteria in cold seep sponge ground. Environ. Microbiome.

[27] Dinghua L., Chi-Man L., Ruibang L., Kunihiko S., Tak-Wah L. (2015). MEGAHIT: An ultra-fast single-node solution for large and complex metagenomics assembly via succinct de Bruijn graph. Bioinformatics.

[28] Hyatt D., LoCascio P.F., Hauser L.J., Uberbacher E.C. (2012). Gene and translation initiation site prediction in metagenomic sequences. Bioinformatics.

[29] Fu L., Niu B., Zhu Z., Wu S., Li W. (2012). CD-HIT: Accelerated for clustering the next-generation sequencing data. Bioinformatics.

[30] Heng L., Richard D. (2010). Fast and accurate short read alignment with Burrows–Wheeler transform. Bioinformatics.

[31] Xie F., Jin W., Si H., Yuan Y., Tao Y., Liu J., Wang X., Yang C., Li Q., Yan X. (2021). An integrated gene catalog and over 10,000 metagenome-assembled genomes from the gastrointestinal microbiome of ruminants. Microbiome.

[32] Buchfink B., Xie C., Huson D.H. (2015). Fast and sensitive protein alignment using DIAMOND. Nat. Methods.

[33] Schloss P.D., Westcott S.L., Ryabin T., Hall J.R., Hartmann M., Hollister E.B., Lesniewski R.A., Oakley B.B., Parks D.H., Robinson C.J. (2009). Introducing mothur: Open-Source, Platform-Independent, Community-Supported Software for Describing and Comparing Microbial Communities. Appl. Environ. Microbiol..

[34] Kang D.D., Li F., Kirton E., Thomas A., Egan R., An H., Wang Z. (2019). MetaBAT 2: An adaptive binning algorithm for robust and efficient genome reconstruction from metagenome assemblies. PeerJ.

[35] Parks D.H., Imelfort M., Skennerton C.T., Hugenholtz P., Tyson G.W. (2015). CheckM: Assessing the quality of microbial genomes recovered from isolates, single cells, and metagenomes. Genome Res..

[36] Olm M.R., Brown C.T., Brooks B., Banfield J.F. (2017). dRep: A tool for fast and accurate genomic comparisons that enables improved genome recovery from metagenomes through de-replication. ISME J..

[37] Chaumeil P.-A., Mussig A.J., Hugenholtz P., Parks D.H. (2019). GTDB-Tk: A toolkit to classify genomes with the Genome Taxonomy Database. Bioinformatics.

[38] Jäckle O., Seah B.K.B., Tietjen M., Leisch N., Liebeke M., Kleiner M., Berg J.S., Gruber-Vodicka H.R. (2019). Chemosynthetic symbiont with a drastically reduced genome serves as primary energy storage in the marine flatworm *Paracatenula*. Proc. Natl. Acad. Sci. USA.

[39] Simister R.L., Deines P., Botté E.S., Webster N.S., Taylor M.W. (2011). Sponge-specific clusters revisited: A comprehensive phylogeny of sponge-associated microorganisms. Environ. Microbiol..

[40] Taylor M.W., Tsai P., Simister R.L., Deines P., Botte E., Ericson G., Schmitt S., Webster N.S. (2012). ‘Sponge-specific’ bacteria are widespread (but rare) in diverse marine environments. ISME J..

[41] Regnier P., Dale A.W., Arndt S., LaRowe D.E., Mogollón J.M., Van Cappellen P. (2011). Quantitative analysis of anaerobic oxidation of methane (AOM) in marine sediments: A modeling perspective. Earth-Sci. Rev..

[42] Cochran J.K., Landman N.H., Jakubowicz M., Brezina J., Naujokaityte J., Rashkova A., Garb M.P., Larson N.L. (2022). Geochemistry of Cold Hydrocarbon Seeps: An Overview. Ancient Hydrocarbon Seeps.

[43] Xiao X., Luo M., Zhang C., Zhang T., Yin X., Wu X., Zhao J., Tao J., Chen Z., Liang Q. (2023). Metal-Driven Anaerobic Oxidation of Methane as an Important Methane Sink in Methanic Cold Seep Sediments. Microbiol. Spectr..

[44] Josep R., Michael H., Elias S.O., Casamayor E.O., Noah F. (2024). Leveraging genomic information to predict environmental preferences of bacteria. ISME J..

[45] Morris J.J., Lenski R.E., Zinser E.R. (2012). The Black Queen Hypothesis: Evolution of Dependencies through Adaptive Gene Loss. mBio.

[46] Chen Y., Neilson J.W., Kushwaha P., Maier R.M., Barberán A. (2020). Life-history strategies of soil microbial communities in an arid ecosystem. ISME J..

[47] Hannes H., Slaby B.M., Jahn M.T., Kristina B., Lucas M.S., Frank F.R., Abdelmohsen U.R., Ute H. (2016). An Enrichment of CRISPR and Other Defense-Related Features in Marine Sponge-Associated Microbial Metagenomes. Front. Microbiol..

[48] Thomas T., Moitinho-Silva L., Lurgi M., Björk J.R., Easson C., Astudillo-García C., Olson J.B., Erwin P.M., López-Legentil S., Luter H. (2016). Diversity, structure and convergent evolution of the global sponge microbiome. Nat. Commun..

[49] Zhang D., Gao F., Jakovlić I., Zhou H., Zhang J., Li W.X., Wang G.T. (2019). PhyloSuite: An integrated and scalable desktop platform for streamlined molecular sequence data management and evolutionary phylogenetics studies. Mol. Ecol. Resour..

